# Genotype‐phenotype correlations and BH_4_ estimated responsiveness in patients with phenylketonuria from Rio de Janeiro, Southeast Brazil

**DOI:** 10.1002/mgg3.610

**Published:** 2019-03-03

**Authors:** Eduardo Vieira Neto, Francisco Laranjeira, Dulce Quelhas, Isaura Ribeiro, Alexandre Seabra, Nicole Mineiro, Lilian M. Carvalho, Lúcia Lacerda, Márcia G. Ribeiro

**Affiliations:** ^1^ Agência Nacional de Saúde Suplementar Gerência de Monitoramento Assistencial Rio de Janeiro Brazil; ^2^ Serviço de Genética Médica Instituto de Puericultura e Pediatria Martagão Gesteira Universidade Federal do Rio de Janeiro Rio de Janeiro Brazil; ^3^ Centro de Genética Médica Doutor Jacinto Magalhães Unidade de Bioquímica Genética Porto Portugal; ^4^ Unidade Multidisciplinar de Investigação Biomédica Universidade do Porto Porto Portugal; ^5^ Instituto de Ciências Biomédicas Abel Salazar Universidade do Porto Porto Portugal; ^6^ Serviço de Metabologia Instituto Estadual de Diabetes e Endocrinologia Luiz Capriglione Rio de Janeiro Brazil

**Keywords:** Brazil, genetic association studies, genotype, hyperphenylalaninemia, phenotype, phenylalanine hydroxylase, phenylketonuria

## Abstract

**Background:**

Genetic heterogeneity and compound heterozygosis give rise to a continuous spectrum of phenylalanine hydroxylase deficiency and metabolic phenotypes in phenylketonuria (PKU). The most used parameters for evaluating phenotype in PKU are pretreatment phenylalanine (Phe) levels, tolerance for dietary Phe, and Phe overloading test. Phenotype can vary from a “classic” (severe) form to mild hyperphenylalaninemia, which does not require dietary treatment. A subset of patients is responsive to treatment by the cofactor tetrahydrobiopterin (BH_4_). Genotypes of PKU patients from Rio de Janeiro, Brazil, were compared to predicted and observed phenotypes. Genotype‐based estimations of responsiveness to BH_4_ were also conducted.

**Methods:**

Phenotype was defined by pretreatment Phe levels. A standard prediction system based on arbitrary assigned values was employed to measure genotype‐phenotype concordance. Patients were also estimated as BH_4_‐responders according to the responsiveness previously reported for their mutations and genotypes.

**Results:**

A 48.3% concordance rate between genotype‐predicted and observed phenotypes was found. When the predicted phenotypes included those reported at the BIOPKU database, the concordance rate reached 77%. A total of 18 genotypes from 30 patients (29.4%) were estimated as of potential or probable BH_4_ responsiveness. Inconsistencies were observed in genotypic combinations including the common “moderate” mutations p.R261Q, p.V388M, and p.I65T and the mild mutations p.L48S, p.R68S, and p.L249F.

**Conclusion:**

The high discordance rate between genotype‐predicted and observed metabolic phenotypes in this study seems to be due partially to the high frequency of the so‐called “moderate” common mutations, p.R261Q, p.V388M, and p.I65T, which are reported to be associated to erratic or more severe than expected metabolic phenotypes. Although our results of BH_4_ estimated responsiveness must be regarded as tentative, it should be emphasized that genotyping and genotype‐phenotype association studies are important in selecting patients to be offered a BH_4_ overload test, especially in low‐resource settings like Brazil.

## 1 INTRODUCTION

Phenylketonuria (PKU; OMIM #261600) is a hereditary autosomal recessive disease characterized by an accumulation of the amino acid phenylalanine (Phe) in blood and other tissues (Donlon, Sarkissian, Levy, & Scriver, 2014). The disease arises from a total or partial deficiency of the activity of the hepatic enzyme phenylalanine hydroxylase (PAH; EC 1.14.16.1), which catalyzes the conversion of Phe to tyrosine (Tyr) in the presence of the cofactor tetrahydrobiopterin (BH_4_) and nonheme iron (Sumaily & Mujamammi, 2017). This deficiency is caused by mutations in the *PAH* gene—OMIM #612349 (Blau, Shen, & Carducci, 2014). PKU incidence varies widely among ethnic groups and countries (Blau et al., 2014). In Latin America (Borrajo, 2007) and Brazil (de Carvalho, dos Santos, dos Santos, Vargas, & Pedrosa, 2007), incidences of 1:21,000 (1:12,000–1:52,000) and 1:25,300 live births were estimated, respectively.

The degree of deficiency of PAH activity is very variable among the affected individuals, which produces a continuous spectrum of metabolic phenotypes. The most used parameters of the metabolic phenotypes are pretreatment Phe levels, tolerance for dietary Phe, and Phe overloading test (Blau et al., 2014). Patients with supposedly absent or near‐absent residual enzyme activity present “classic” (severe) PKU, which is characterized by a pretreatment blood Phe level ≥1,200 μmol/L (≥20 mg/dl) and a tolerance for dietary Phe of <20 mg/kg of body weight/day—250–350 mg/day (Guldberg et al., 1998; Michals‐Matalon, Bhatia, Guttler, Tyring, & Matalon, 2007; Mitchell et al., 2005). The milder forms of PKU can be arbitrarily subdivided into moderate PKU (pretreatment Phe 900–1,200 μmol/L, 15–20 mg/dl) and mild PKU (pretreatment Phe 600–900 μmol/L, 10–15 mg/dl). Patients with moderate PKU present a tolerance for dietary Phe of 20–25 mg/kg of body weight/day (350–400 mg/day) and those with mild PKU tolerate 25–50 mg/kg of body weight/day (400–600 mg/day). Individuals who maintain levels of Phe in the range of 300–600 μmol/L (5–10 mg/dl) on a normal diet are classified as having mild hyperphenylalaninemia (MHP). These individuals generally do not need diet therapy.

Genotypically, PKU is also very diverse. More than 1,000 *PAH* gene variants are tabulated in the *PAH*vdb—Phe Hydroxylase Gene Locus‐Specific Database (http://www.biopku.org/pah/search-results-browse.asp), of which circa 630 are disease‐causing mutations (BIOPKU; http://www.biopku.org/biopku/search-start.asp). In the PKU population of Rio de Janeiro, our group formerly found 37 causative mutations, among a majority (91.2%) of compound heterozygotes (Vieira Neto et al., 2018). Missense variants, variants at splicing sites, in‐frame deletions, deletions with frameshift, nonsense variants, and large deletions corresponded to 63.7%, 22.6%, 5.4%, 3.9%, 2.9%, and 1.5% of the mutant alleles, respectively (Vieira Neto et al., 2018).

Genotype‐phenotype correlation studies in PKU/MHP traditionally employ predicted PAH residual activity (PRA) of each mutation provided by in vitro experiments or by the analysis of its effect on protein structure (Danecka et al., 2015; Gjetting, Petersen, Guldberg, & Guttler, 2001; Pey, Desviat, Gamez, Ugarte, & Perez, 2003; Pey, Stricher, Serrano, & Martinez, 2007; Trunzo et al., 2016). Another approach for investigating genotype‐phenotype correlations is the analysis both of homoallelic mutant genotypes and of the so‐called “functionally hemizygous” heteroallelic genotypes, that is, patients who carry a functionally null mutation on one of their alleles and a missense or an in‐frame small deletion on the other (Kayaalp et al., 1997). Guldberg et al. (1998) proposed a scale to evaluate genotype‐phenotype correlations by the severity of mutant alleles assigning them an arbitrary assigned value (AV) following this scheme: AV = 1, classic PKU; AV = 2, moderate PKU; AV = 4, mild PKU; AV = 8, MHP. The resultant sum of the two scores was correlated by the authors to the patient's phenotype.

Some mutations, usually found in mild to moderate PKU patients and presenting a significant PRA, are associated with BH_4_‐responsive PKU (Blau, 2016; Blau et al., 2014; Zurfluh et al., 2008).

In this study, the genotypes of PKU patients from Rio de Janeiro (Vieira Neto et al., 2018) were correlated to phenotypic severity, to Guldberg et al. (1998) scoring system and to pretreatment Phe levels. Genotype‐based estimations of responsiveness to BH_4_, and the phenotypes of homoallelic and functionally hemizygous heteroallelic genotypes were also analyzed.

## 2 MATERIALS AND METHODS

### 2.1 Subjects

Patients from a PKU metabolic center in Rio de Janeiro, Southeast Brazil, were invited to participate in this study. Of the approximately 150 patients with PKU followed in the reference center's outpatient clinics, a total of 102 patients from 95 families accepted to participate in this study. Ethical approval was obtained from the National Research Ethics Commission of Brazil. The study was performed in accordance with the Guidelines and Standards for Research in Human Beings, established by the Brazilian National Health Council (Brazil's Ministry of Health. National Health Council, 2013). Written informed consent was obtained from each adult patient and parent or guardian of each child, adolescent and intellectually disabled patient enrolled in this study. Seven were late‐treated PKU patients (diagnosis of PKU of any severity phenotype after 120 days of age), and the rest (95) were early‐treated patients detected by newborn screening. A total of 98 patients were assigned to one of the following four phenotypes according to pretreatment Phe levels in a recall sample after a positive newborn screening sample: classic PKU, ≥1,200 μmol/L (≥20 mg/dl); moderate PKU, ≥900 μmol/L and <1,200 μmol/L (≥15 and <20 mg/dl); mild PKU, ≥600 μmol/L and <900 μmol/L (≥10 and <15 mg/dl); MHP, ≥360 and <600 μmol/L (≥6 and <10 mg/dl). Four patients were allocated to one of these phenotypes as indicated by the evaluation of a physician and a nutritionist.

### 2.2 Genotype analysis

All patients were completely genotyped formerly by our group (Vieira Neto et al., 2018). Table 1 presents the mutations found in these patients and their predicted PAH residual activities (null mutations pointed out) as indicated by Himmelreich et al. (2018), from previous publications (Aldamiz‐Echevarria et al., 2016; Eisensmith et al., 1996; Guldberg et al., 1998; Jeannesson‐Thivisol et al., 2015; Pey et al., 2003; Trunzo et al., 2016) or judged as null in the case of nonsense mutations, frameshift and large deletions whose PAH residual activities were not formerly reported.

**Table 1:** Causative mutations in the *PAH* gene found in patients participating in this study, their predicted phenylalanine hydroxylase (PAH) residual activity according to Himmelreich et al. (2018), arbitrary assigned values (AV) of Guldberg et al. (1998), and estimated BH_4_ responsiveness as listed in the BIOPKU database and stated by Zurfluh et al. (2008)

Mutation		Mutation type	PAH residual activity (%)^a^	AV^b^	Estimated BH_4_ responsiveness	
Nucleotide change	Amino acid change (trivial name)				BIOPKU database	Zurfluh et al. (2008)
c.1055delG	p.G352Vfs*48	Frameshift Deletion	0^e^	1^d^	NR^r^	UNK
c.165delT	p.F55Lfs*6	Frameshift Deletion	0^f^	1	NR^s^	UNC
c.503delA	p.Y168Sfs*27	Frameshift Deletion	0^e^	1^d^	NR^t^	UNK
c.116_118delTCT	p.F39del	In‐frame Deletion	20^c^^,^ z	1^g^	NR^u^	R
c.967_969delACA	p.T323del	In‐frame Deletion	UNK	1^h^	UNK	UNK
c.442‐?_509+?del	?	Large Deletion	0^e^	1^d^	UNK	UNK
c.1042C>G	p.L348V	Missense	32^c^	2	R	R
c.1045T>C	p.S349P	Missense	0^c^^,^ f	1	NR	NR
c.1162G>A	p.V388M	Missense	47^c^	2	R	R
c.1222C>T	p.R408W	Missense	2^c^^,^ f	1	NR	NR
c.1223G>A	p.R408Q	Missense	53^c^	4	R	R
c.1241A>G	p.Y414C	Missense	53^c^	4	R	R
c.1243G>A	p.D415N	Missense	72^c^	8	R	R
c.136G>A	p.G46S	Missense	16^y^	UNC^j^	R	NR^q^
c.143T>C	p.L48S	Missense	47^c^	4	R	R
c.194T>C	p.I65T	Missense	33^c^	2	R	R
c.204A>T	p.R68S	Missense	61^c^	4	R	R
c.250G>T	p.D84Y	Missense	UNK	UNK	UNK	UNK
c.473G>A	p.R158Q	Missense	13^c^	1	NR^w^	R
c.561G>C	p.W187C	Missense	1^f^	1	UNK	UNK
c.745C>T	p.L249F	Missense	51^o^	4^o^	NR	NR^o^
c.754C>T	p.R252W	Missense	0^c^^,^ f	1	NR	NR
c.782G>A	p.R261Q	Missense	34^c^	2	R	R
c.809G>A	p.R270K	Missense	11^o^	1^o^	NR	NR^o^
c.842C>T	p.P281L	Missense	0^c^^,^ f	1	NR	NR
c.934G>T	p.G312C	Missense	New mutation^p^	New mutation^p^	New mutation^p^	New mutation^p^
c.994G>A	p.G332R	Missense	UNK	UNK	UNK	UNK
c.498C>G	p.Y166*	Nonsense	0^e^	1^d^	NR^l^	UNK
c.526C>T	p.R176*	Nonsense	0^f^	1	NR	NR
c.618C>G	p.Y206*	Nonsense	0^f^	1	UNK	UNK
c.781C>T	p.R261*	Nonsense	0^f^	1	NR	NR
c.1066‐11G>A	p.Q355_Y356insGLQ (IVS10‐11G>A)	Splicing	0^c^	1	NR	UNC
c.1199+17G>A	? (IVS11+17G>A)	Splicing	UNK	UNK	UNK	UNK
c.1315+1G>A	? (IVS12+1G>A)	Splicing	0^f^	1	NR	NR
c.168+5G>C	? (IVS2+5G>C)	Splicing	0^f^	1	NR	UNC^k^
c.441+5G>T	? (IVS4+5G>T)	Splicing	UNK	1	NR	UNC^m^
c.842+1G>A	? (IVS7+1G>A)	Splicing	0^f^	1	NR	UNC^n^

*Note*.

NR: non‐responsive; R: responsive; UNC: uncertain; UNK: unknown.

*Stop codon.

^a^Compared with the wild‐type activity. ^b^AV = 1, for classic‐PKU mutations; AV = 2, for moderate‐PKU mutations; AV = 4, for mild‐PKU mutations; and AV = 8, for MHP mutations. ^c^Himmelreich et al. (2018), preferably average of data obtained from in vitro studies of PAH activity in COS cells systems, whenever available. ^d^Estimation of the authors: AV = 1 regarding nonsense mutations, frameshift and large deletions whose PAH residual activities were not formerly reported. ^e^Estimated by the authors as a null mutation. ^f^Formerly reported as a null mutation by Guldberg et al. (1998), Pey et al. (2003, 2007), Eisensmith et al. (1996), and Jeannesson‐Thivisol et al. (2015). ^g^Based on data from 26 patients in the BIOPKU database, 18 homozygotes and eight compound heterozygotes with a null mutation. ^h^Based on data from five patients in the BIOPKU database, three homozygotes, and two compound heterozygotes with a null mutation. ^i^Zurfluh et al. (2008) consider this mutation as of uncertain BH_4_ responsiveness but in the BIOPKU database of the 107 homozygous patients tested, 101 did not respond and six responded slowly. ^j^p.G46S is reported as “mild,” “severe‐mild,” or “severe” by different authors (Pey et al., 2003). ^k^Zurfluh et al. (2008) consider this mutation as of uncertain BH_4_ responsiveness but in the BIOPKU database all three homozygous patients tested did not respond. ^l^Based on data from two homozygous patients in the BIOPKU database. ^m^Zurfluh et al. (2008) consider this mutation as of uncertain BH_4_ responsiveness but data from 21 homozygous patients in the BIOPKU database, which presented classic PKU, showed that all those tested (12) were BH_4_ nonresponsive. ^n^Zurfluh et al. (2008) consider this mutation as of uncertain BH_4_ responsiveness but data from nine patients in the BIOPKU database, two homozygotes and seven compound heterozygotes with a null mutation, showed they were BH_4_ nonresponsive, although two compound heterozygotes with a null mutation were slow responders. ^o^Data from Trunzo et al. (2016). ^p^Described by our group (Vieira Neto et al., 2018). ^q^Data from Leandro, Simonsen, Saraste, Leandro, and Flatmark (2011) report it as nonreponsive. ^r^Based on data from 29 homozygous patients in the BIOPKU database, which presented classic PKU, and those tested (18) were BH_4_ nonresponsive. ^s^Based on data from 24 compound heterozygotes with a null mutation in the BIOPKU database that were tested, all of which were BH_4_ nonresponsive. ^t^In the BIOPKU database, one compound heterozygote with a null mutation was BH_4_ nonresponsive. ^u^One homozygous patient and five compound heterozygotes with a null mutation reported by Aldamiz‐Echevarria et al. (2016) and two compound heterozygotes with a null mutation reported by Jeannesson‐Thivisol et al. (2015) were BH_4_ nonresponsive. ^v^In the BIOPKU database, one compound heterozygote with a null mutation was BH_4_ responsive. ^w^Based on data from 13 homozygous patients in the BIOPKU database that were tested, 12 of which were BH_4_ nonresponsive. ^x^Expression in *Escherichia coli*. ^y^Pey et al. (2007). ^z^Expression in TNT‐T7.

### 2.3 Genotype–phenotype correlations and genotype‐based estimations of BH_4_‐responsiveness

The predicted PRA was calculated for each genotypic combination as an average from data compiled in Table 1. A phenotype prediction system based on arbitrary assigned values (AV) according to Guldberg et al. (1998) was employed to compare expected and observed phenotypes: AV = 1 was assigned to mutations that result in a nonfunctional PAH enzyme phenotype (null mutations), including nonsense (introduction of a premature stop codon), most splice site mutations affecting the invariant AG‐GT dinucleotides, small deletions with frameshift, some in‐frame deletions, large indels, and missense mutations that an in vitro expression analysis demonstrated an enzyme activity typically below <3% or <1% of normal (Kayaalp et al., 1997)—these mutations when in homozygosis or in compound heterozygosis with another null mutation produce typically a classic PKU phenotype; AV = 2, for moderate PKU mutations; AV = 4, for mild PKU mutations; and AV = 8 for MHP mutations. The resulting sums of the AVs of the genotypic combinations were interpreted according to Guldberg et al. (1998) method, which is summarized here: AV sum = 2, classic PKU; AV sum = 3, moderate PKU; AV sum = 4, moderate/mild PKU; AV sum = 5 or 6, mild PKU; AV sum = 8, mild PKU/MHP; AV sum ≥ 9, MHP.

Estimated BH_4_ responsiveness of each mutation as a categorical variable (“Yes,” “No,” “Unclear” or “Unknown”) was based on the work of Zurfluh et al. (2008) and as indicated when analyzing the BIOPKU database (http://www.biopku.org). A mutation was considered by Zurfluh et al. (2008) as associated with BH_4_‐responsiveness if it was present either in homozygosis or in compound heterozygosis with a known null mutation in patients that were classified as BH_4_‐responsive—response to the oral administration of BH_4_ (10–20 mg/kg body weight) by lowering their blood Phe levels by at least 30% within 8–24 hr. In the BIOPKU database, the indication of BH_4_‐responsiveness of a mutation was taken into account when the majority of listed patients carrying it either in homozygosis or in compound heterozygosis with a known null mutation was classified as BH_4_‐responsive. Table 1 specifies the AV and BH_4_ responsiveness of each mutation by these two criteria in our patients. Patients were assigned as candidates for BH_4_ testing according to the criteria of Vela‐Amieva et al. (2015): (a) genotypic combination previously reported as responsive by Aldamiz‐Echevarria et al. (2016), Jeannesson‐Thivisol et al. (2015), Scala et al. (2015), Couce et al. (2013), Karacic et al. (2009), or in the BIOPKU database (http://www.biopku.org); (b) at least one mutant allele assigned as responsive by Zurfluh et al. (2008) or in the BIOPKU database; and (c) those patients whose genotypic combination has no documented evidence of responsiveness/nonresponsiveness.

### 2.4 “Functionally hemizygous” genetic combinations

The phenotypes of functionally hemizygous (Guldberg et al., 1998) patients, that is, patients who carry a functionally null mutation on one of their alleles, were used to evaluate the effect of missense alleles in some patients.

### 2.5 Statistics

The statistical software package Stata/SE 12.1 for Mac (StataCorp) was employed to analyze the data. The relationship between the predicted PAH residual activities and pretreatment Phe levels was evaluated by linear regression and Pearson's correlation coefficient. A two‐sample test of proportions was used to compare genotype‐phenotype discordance of genotypes containing p.R261Q, p.V388M, or p.I65T mutations with the discordance of other genotypes.

## 3 RESULTS

### 3.1 Mutation and phenotype distributions

A total of 77 genotypic combinations were observed in our sample. Most of the genotypes (58/77, 75.3%) were found in just one patient (Table 2).

**Table 2:** Genotypes, observed phenotypes, predicted phenotypes, estimated genotype BH_4_ responsiveness, assignment as BH_4_ test candidate, and genotype‐phenotype inconsistencies of 102 phenylketonuria (PKU)/MHP patients from Rio de Janeiro, Southeast Brazil

Number of patients (relative frequency %)	Genotype [allele 1];[allele 2]	Observed phenotypes^a^ (N)				AV sum	Predicted phenotypes BIOPKU database^b^	Predicted phenotypes AV sum^c^	Estimated genotype BH_4_ responsiveness^d^			BH_4_ test candidate	Inconsistencies		
		cPKU	moPKU	miPKU	MHP				Allele 1	Allele 2	Genotype		BIOPKU	AV sum	Both^f^
4 (3.92)	p.[S349P];[V388M]	1	2	1	—	3	cPKU	moPKU	N	Y	UNC^e^	Y	3	2	1
3 (2.94)	c.[168+5G>C];p.[Q355_Y356insGLQ]	3	—	—		2	cPKU	cPKU	N	N	N	N	0	0	0
3 (2.94)	p.[R261Q];[Q355_Y356insGLQ]	2	1	—	—	3	cPKU/miPKU^g^	moPKU	Y	N	UNC	Y	0	2	0
3 (2.94)	p.[R261Q];[S349P]	1	2	—	—	3	cPKU/miPKU	moPKU	Y	N	UNC	Y	0	1	0
3 (2.94)	p.[R261Q];[R261Q]	1	1	1	—	4	cPKU/miPKU	moPKU/miPKU	Y	Y	UNC	Y	0	1	0
2 (1.96)	c.[168+5G>C];p.[V388M]	1	—	1	—	3	miPKU	moPKU	N	Y	UNK	Y	1	2	1
2 (1.96)	p.[F39del];[V388M]	2	—	—	—	3	—	moPKU	N	Y	UNK	Y	—	2	2
2 (1.96)	p.[W187C];[V388M]	—	1	1	—	3	—	moPKU	UNK	Y	UNK	Y	—	1	1
2 (1.96)	p.[R252W];[V388M]	1	—	1	—	3	cPKU	moPKU	N	Y	N	Y	1	2	1
2 (1.96)	p.[P281L];[V388M]	2	—	—	—	3	cPKU/miPKU	moPKU	N	Y	N	Y	0	2	0
2 (1.96)	p.[T323del];[V388M]	1	1	—	—	UNK^e^	cPKU	UNK	UNK	Y	UNK	Y	1	UNK	1
2 (1.96)	p.[G352Vfs*48];[Q355_Y356insGLQ]	2	—	—	—	2	cPKU	cPKU	N	N	N	N	0	0	0
2 (1.96)	p.[P281L];[Q355_Y356insGLQ]	2	—	—	—	2	cPKU	cPKU	N	N	N	N	0	0	0
2 (1.96)	p.[I65T];[R261Q]	—	1	1	—	4	cPKU/miPKU	moPKU/miPKU	Y	Y	UNC	Y	0	0	0
2 (1.96)	p.[I65T];c.[1315+1G>A]	2	—	—	—	3	cPKU/miPKU	moPKU	Y	N	UNC	Y	0	2	0
2 (1.96)	p.[G312C];c.[1315+1G>A]	—	1	1	—	—h	—	—h	UNK	N	UNK	Y	—	—h	—h
2 (1.96)	p.[R252W];[R261Q]	—	—	2	—	3	cPKU/miPKU	moPKU	N	Y	N	Y	0	2	0
2 (1.96)	p.[L348V];[S349P]	1	1	—	—	3	—	moPKU	Y	N	UNK	Y	—	1	1
2 (1.96)	p.[R261Q];[Y414C]	—	—	2	—	6	miPKU/MHP	miPKU	Y	Y	Y	Y	0	0	0
1 (0.98)	p.[L348V];[V388M]	—	1	—	—	4	cPKU/miPKU	moPKU/miPKU	Y	Y	UNC	Y	0	0	0
1 (0.98)	p.[R261Q];[V388M]	—	—	1	—	4	cPKU/miPKU	moPKU/miPKU	Y	Y	UNC	Y	0	0	0
1 (0.98)	p.[V388M];[V388M]	1	—	—	—	4	cPKU/miPKU	moPKU/miPKU	Y	Y	UNC	Y	0	1	0
1 (0.98)	p.[G332R];[V388M]	1	—	—	—	UNK	—	UNK	UNK	Y	UNK	Y	—	UNK	UNK
1 (0.98)	p.[G46S];[V388M]	1	—	—	—	UNC	—	UNC	Y	Y	UNK	Y	—	UNC	UNC
1 (0.98)	p.[Q355_Y356insGLQ];[V388M]	—	1	—	—	3	cPKU/miPKU	moPKU	N	Y	N^j^	Y	0	0	0
1 (0.98)	p.[R176*];[V388M]	1	—	—	—	3	cPKU/miPKU	moPKU	N	Y	UNK	Y	0	1	0
1 (0.98)	p.[R270K];[V388M]	—	1	—	—	3	—	moPKU	N	Y	UNK	Y	—	0	0
1 (0.98)	p.[R158Q];[V388M]	1	—	—	—	3	cPKU	moPKU	N	Y	N	Y	0	1	0
1 (0.98)	p.[G46S];c.[442‐?_509+?del]	—	1	—	—	UNC	—	UNC	Y	UNK	UNK	Y	—	UNC	UNC
1 (0.98)	c.[442‐?_509+?del];p.[R261Q]	1	—	—	—	3	cPKU	moPKU	UNK	Y	UNK	Y	0	1	0
1 (0.98)	c.[441+5G>T];[442‐?_509+?del]	—	1	—	—	2	—	cPKU	N	UNK	UNK	Y	—	1	1
1 (0.98)	p.[Y166*];[Y166*]	1	—	—	—	2	cPKU	cPKU	N	N	N	N	0	0	0
1 (0.98)	p.[Y168Sfs*27];[Y168Sfs*27]	1	—	—	—	2	—	cPKU	N	N	UNK	Y	—	0	0
1 (0.98)	p.[F55Lfs*6];[R261*]	1	—	—	—	2	cPKU	cPKU	N	N	N	N	0	0	0
1 (0.98)	p.[R252W];[R270K]	1	—	—	—	2	—	cPKU	N	N	UNK	Y	—	0	0
1 (0.98)	p.[R176*];[R252W]	1	—	—	—	2	—	cPKU	N	N	UNK	Y	—	0	0
1 (0.98)	p.[I65T];[S349P]	1	—	—	—	3	cPKU/miPKU	moPKU	Y	N	N	Y	0	1	0
1 (0.98)	c.[168+5G>C];p.[S349P]	1	—	—	—	2	—	cPKU	N	N	UNK	Y	—	0	0
1 (0.98)	p.[R252W];[S349P]	1	—	—	—	2	cPKU	cPKU	N	N	UNK	Y	0	0	0
1 (0.98)	p.[T323del];[S349P]	1	—	—	—	2	—	cPKU	UNK	N	UNK	Y	—	0	0
1 (0.98)	p.[Q355_Y356insGLQ];[Q355_Y356insGLQ]	1	—	—	—	2	cPKU	cPKU	N	N	N	N	0	0	0
1 (0.98)	p.[L348V];[Q355_Y356insGLQ]	—	1	—	—	3	cPKU	moPKU	Y	N	N	Y	1	0	0
1 (0.98)	p.[I65T];[Q355_Y356insGLQ]	—	—	1	—	3	cPKU/miPKU	moPKU	Y	N	UNC	Y	0	1	0
1 (0.98)	p.[D84Y];[Q355_Y356insGLQ]	—	1	—	—	UNK	—	UNK	UNK	N	UNK	Y	—	UNK	UNK
1 (0.98)	p.[L48S];[Q355_Y356insGLQ]	1	—	—	—	5	cPKU/miPKU	miPKU	Y	N	UNC	Y	0	1	0
1 (0.98)	p.[L249F];[Q355_Y356insGLQ]	—	1	—	—	5	—	miPKU	N	N	N	N	—	1	1
1 (0.98)	p.[T323del];[Q355_Y356insGLQ]	1	—	—	—	2	cPKU	cPKU	UNK	N	UNK	Y	0	0	0
1 (0.98)	p.[L249F];[R261Q]	—	—	1	—	6	—	miPKU	N	Y	UNK	Y	—	0	0
1 (0.98)	c.[168+5G>C];[168+5G>C]	1	—	—	—	2	cPKU	cPKU	N	N	N	N	0	0	0
1 (0.98)	p.[Q355_Y356insGLQ];c.[1199+17G>A]	—	1	—	—	UNK	—	UNK	N	UNK	UNK	Y	—	UNK	UNK
1 (0.98)	p.[D84Y];c.[1199+17G>A]	—	—	1	—	UNK	—	UNK	UNK	UNK	UNK	Y	—	UNK	UNK
1 (0.98)	c.[168+5G>C];[1199+17G>A]	—	—	—	1	UNK	—	UNK	N	UNK	UNK	Y	—	UNK	UNK
1 (0.98)	p.[R252W];c.[1199+17G>A]	—	—	—	1	UNK	—	UNK	N	UNK	UNK	Y	—	UNK	UNK
1 (0.98)	p.[Q355_Y356insGLQ];[Y414C]	—	1	—	—	5	miPKU	miPKU	N	Y	UNC	Y	0	1	0
1 (0.98)	p.[R261Q];[R408W]	1	—	—	—	3	cPKU/miPKU	moPKU	Y	N	N^k^	Y	0	1	0
1 (0.98)	p.[R408W];[R408Q]	—	—	1	—	5	miPKU	miPKU	N	Y	Y	Y	0	0	0
1 (0.98)	p.[R158Q];[Y206*]	1	—	—	—	2	—	cPKU	N	UNK	UNK	Y	—	0	0
1 (0.98)	p.[R158Q];[L348V]	1	—	—	—	3	miPKU	moPKU	N	Y	UNK	Y	1	1	1
1 (0.98)	p.[R158Q];[R158Q]	—	1	—	—	2	cPKU/miPKU	cPKU	N	N	N^l^	N	0	1	0
1 (0.98)	p.[R252W];[L348V]	1	—	—	—	3	cPKU	moPKU	N	Y	UNC	Y	0	1	0
1 (0.98)	p.[R252W];[P281L]	—	1	—	—	2	cPKU	cPKU	N	N	N	N	1	1	1
1 (0.98)	c.[168+5G>C];p.[P281L]	—	1	—	—	2	cPKU	cPKU	N	N	UNK	Y	1	1	1
1 (0.98)	c.[168+5G>C];p.[L249F]	1	—	—	—	5	—	miPKU	N	N	UNK	Y	—	1	1
1 (0.98)	p.[R261Q];[G352Vfs*48]	—	—	1	—	3	cPKU	moPKU	Y	N	N	Y	1	1	1
1 (0.98)	p.[G352Vfs*48];[D415N]	—	—	1	—	9	—	MHP	N	Y	UNK	Y	—	1	1
1 (0.98)	c.[168+5G>C];p.[D415N]	—	—	—	1	9	—	MHP	N	Y	UNK	Y	—	0	0
1 (0.98)	p.[R252W];[Y414C]	—	1	—	—	5	miPKU	miPKU	N	Y	Y	Y	0	1	0
1 (0.98)	p.[G352Vfs*48];[Y414C]	—	—	1	—	5	—	miPKU	N	Y	UNK	Y	—	0	0
1 (0.98)	p.[I65T];[R68S]	1	—	—	—	6	miPKU	miPKU	Y	Y	UNC	Y	1	1	1
1 (0.98)	p.[I65T];c.[842+1G>A]	1	—	—	—	3	cPKU	moPKU	Y	N	N	Y	0	1	0
1 (0.98)	p.[R68S];c.[1315+1G>A]	1	—	—	—	5	miPKU	miPKU	Y	N	UNC	Y	1	1	1
1 (0.98)	p.[L249F];c.[1315+1G>A]	1	—	—	—	5	—	miPKU	N	N	UNK	Y	—	1	1
1 (0.98)	p.[I65T];[T323del]	1	—	—	—	3	—	moPKU	Y	UNK	UNK	Y	—	1	1
1 (0.98)	p.[D84Y];[T323del]	—	—	1	—	UNK	—	UNK	UNK	UNK	UNK	Y	—	UNK	UNK
1 (0.98)	c.[168+5G>C];p.[T323del]	1	—	—	—	2	—	cPKU	N	UNK	UNK	Y	—	0	0
1 (0.98)	p.[P281L];[T323del]	1	—	—	—	2	—	cPKU	N	UNK	UNK	Y	—	0	0
1 (0.98)	p.[R261Q];[T323del]	1	—	—	—	3	—	moPKU	Y	UNK	UNK	Y	—	1	1

*Stop codon.

^a^Based on pretreatment phenylalanine levels: cPKU: classic PKU; moPKU: moderate PKU; miPKU: mild PKU; MHP: mild hyperphenylalaninemia. ^b^BIOPKU database (http://www.biopku.org). ^c^AV sum method of Guldberg et al. (1998). ^d^According to Aldamiz‐Echevarria et al. (2016), Jeannesson‐Thivisol et al. (2015), Scala et al. (2015), Couce et al. (2013), Karacic et al. (2009), or in the BIOPKU database. ^e^UNK = unknown; UNC = uncertain; Y = yes; N = no. ^f^Both the phenotypes predicted by AV Sum or reported at the BIOPKU database were regarded as consistent with the genotypic combination. ^g^BIOPKU database mild PKU category was considered as representing both moderate and mild PKU. ^h^New genotypic combination. iThe number of patients tested for BH_4_ responsiveness was less than 30% of the total of patients with this genotype at the BIOPKU database. ^j^One (11,1%) patient reported by Aldamiz‐Echevarria et al. (2016) as responsive out of a total of nine patients from the same authors, BIOPKU database, and Jeannesson‐Thivisol et al. (2015). ^k^Two (4,3%) patients reported at the BIOPKU database as responsive out of a total of 46 patients from this same database, Aldamiz‐Echevarria et al. (2016), and Jeannesson‐Thivisol et al. (2015). ^l^One (6.7%) patient reported at the BIOPKU database as responsive out of a total of 15 patients from this same database, Aldamiz‐Echevarria et al. (2016), and Jeannesson‐Thivisol et al. (2015).

Of the 98 patients whose pretreatment Phe levels could be retrieved, 51 (52.0%) were classified as classic PKU, 25 (25.5%) as moderate PKU, 19 (19.4%) as mild PKU, and three (3.1%) as MHP. Three classic and one mild PKU patients, with no pretreatment Phe levels data, were classified according to an evaluation by a physician and a nutritionist.

### 3.2 The AV phenotype prediction system

In our sample, nine patients were homoallelic. Four of the five patients homoallelic for severe or null mutations (p.Q355_Y356insGLQ, formerly IVS10‐11G>A, p.Y166*, c.168+5G>C, and p.Y168Sfs*27) exhibited a classic phenotype in accordance to the AV system. However, one patient homoallelic for the p.R158Q mutation had a moderate phenotype. On the other hand, two of the four homoallelic patients for mutations with PRA ≥12%, p.V388M and p.R261Q, which by the AV system should present a moderate/mild phenotype, had a classic phenotype—discordance of 50% (Table 2).

Among the heteroallelic genotypes, 16 (20 patients) were combinations of null or severe mutations, 37 (52 patients) were “functionally hemizygous” genotypes and six genotypes (eight patients) were combinations of two mutations with AV value ≥ 2 (Table 2).

In summary, the analysis of the 66 genotypes with information on AV, comprising 89 patients, demonstrated that the observed phenotype matched the AV predicted phenotype in 48.3% of the cases (discordance rate of 51.7%). If the phenotypes reported for the genotypic combinations in BIOPKU database were included as consistent outcomes, the discordance rate fell to 23.1% (Table 2). Moreover, only five patients (5.6%) had an observed phenotype more than one category away from that predicted. Table 3 summarizes the results of the AV phenotypic prediction system.

**Table 3:** Observed phenotypes based on pretreatment phenylalanine levels (85 patients) or clinical evaluation (four patients) versus expected phenotypes according to the sum of the arbitrary assigned values (AV) for each genotype, according to the phenotype prediction system of Guldberg et al. (1998) for patients with PKU or mild hyperphenylalaninemia (MHP) from Rio de Janeiro, Brazil

AV sum	Expected phenotype	Observed phenotype				Total (expected)	Perfect matches, %
		Classic	Moderate	Mild	MHP		
2	Classic	21	4			25	84.0
3	Moderate	23	10	8		41	24.4
4	Moderate/mild	2	3	3		8	75.0
5 and 6	Mild	5	3	5		13	38.5
9	MHP			1	1	2	50.0
Total (observed)		51	20	17	1	89	48.3

Analyzing the genotype‐phenotype discordance in functionally hemizygous patients, we found that patients carrying the common moderate mutations p.R261Q, p.V388M, and p.I65T presented a high level of discordant phenotypes, usually more severe than expected. A total of 18 out of 34 functionally hemizygous patients (53.0%) exhibited a classic, and 8 (23.5%) a mild phenotype. Only eight (23.5%) presented the expected moderate phenotype. Inconsistencies were also observed in genotypes involving a severe or null mutation and the mild mutations p.L48S, p.R68S, and p.L249F. None of the five patients presented the expected mild phenotype—four exhibited a classic and one a moderate phenotype.

### 3.3 Selected new genotypes

The genotypes involving the mutation c.1199+17G>A (rs62508613), p.[Q355_Y356insGLQ];c.[1199+17G>A], p.[D84Y];c.[1199+17G>A], c.[168+5G>C];[1199+17G>A], p.[R252W];c.[1199+17G>A], were found in two moderate/mild PKU and two MHP patients. Three of these patients were functionally hemizygous patients.

### 3.4 Predicted PRA and pretreatment Phe levels

**Figure 1:**
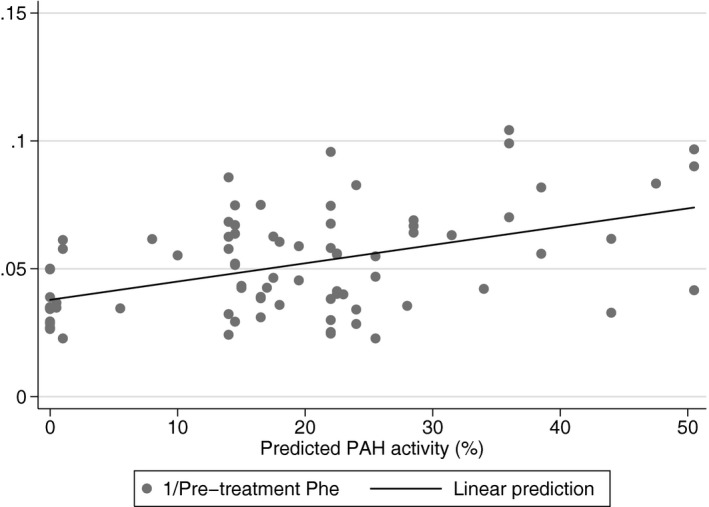
Comparison of the inverse of pretreatment phenylalanine (Phe) levels (mg/dl) with the predicted phenylalanine hydroxylase (PAH) enzyme activity for a total of 81 patients for which mutant PAH residual activity (PRA) for both alleles and pretreatment Phe levels were both known. Mean PRA for each genotype was established by data in BIOPKU database and previously published articles (Aldamiz‐Echevarria et al., 2016; Eisensmith et al., 1996; Guldberg et al., 1998; Jeannesson‐Thivisol et al., 2015; Pey et al., 2003; Trunzo et al., 2016). A significant correlation (*t* = 4.79, *p* < 0.0001) was found by linear regression analysis: *y* = 0.0007*x* + 0.0379, *R* ^2^ = 0.225

A strong relationship between mutation severity, according to the level of PRA, and the inverse of pretreatment Phe levels was observed (*t* = 4.79, *p* < 0.0001)—Figure 1. Means (±*SD*) of PRA activity of classic, moderate, and mild PKU groups were 13.9% (12.4), 19.8% (11.1), and 29.1% (13.4), respectively. There was a statistically significant difference in PRA among these severity groups as determined by one‐way ANOVA (*F*(2, 81) = 9.72, *p* = 0.0002). A Tukey test revealed that PRA was statistically significantly higher in the mild group compared to the classic group (*p* < 0.0001). However, there were no statistically significant differences between classic and moderate groups (*p* = 0.176), and between moderate and mild groups (*p* = 0.063).

### 3.5 BH_4_ estimated responsiveness

Ten (27.0%) out of the 37 mutations found in our population were previously reported as BH_4_ responsive, 19 (51.4%) as nonresponsive. The BH_4_‐responsiveness of 7 (18.9%) mutations has not been established up to this time, and one mutation was first described by our group (Vieira Neto et al., 2018)—Table 1.

Twelve patients (11.8%), corresponding to eight genotypes, carried two responsive mutations. A total of 54 patients (52.9%), corresponding to 38 genotypes, carried one responsive mutation. Thus, 66 patients (64.7%), distributed among 46 genotypes, had at least one responsive mutation.

Fifteen genotypes, with at least one responsive allele, from 26 patients (25.5%) were potentially responsive to BH_4_—uncertain responsiveness to BH_4_ in Table 2. Three genotypes, with at least one responsive allele, from four patients (3.9%), had an extremely high probability of responsiveness to BH_4_—Y in Table 2. Ten genotypes from 13 patients (12.7%), with just one responsive allele, were estimated not to respond to BH4—N in Table 2. The BH_4_ responsiveness status of 18 genotypes, from 23 patients (22.5%) carrying at least one responsive allele, could not be estimated—unknown responsiveness to BH_4_ in Table 2.

Eighteen genotypes, distributed among 22 patients (21.6%), were combinations of BH_4_ nonresponsive alleles. Ten of these genotypes, from 14 patients (13.7%), were estimated to be BH_4_ nonresponsive—Table 2. Eight genotypes from eight patients (7.8%), with two BH_4_ nonresponsive alleles, although probably also nonresponsive, as their BH_4_ responsiveness has not been reported yet, were considered as genotypes of unknown responsiveness to BH_4_—Table 2.

Eleven genotypes from 12 patients (11.8%) were combinations of a nonresponsive allele with an allele of unknown responsiveness to BH_4_—they were considered as genotypes of unknown responsiveness to BH_4_—Table 2. Two genotypes from two patients (2.0%) were combinations of alleles of unknown responsiveness to BH_4_.

In summary, 18 genotypes from 30 patients (29.4%) were estimated as genotypic combinations of potential or probable BH_4_ responsiveness; 28 genotypes from 35 patients (34.3%) were estimated as genotypes of improbable responsiveness to BH_4_; 31 genotypes from 37 patients (36.3%) were considered as genotypic combinations of unknown responsiveness to BH_4_.

Only 14 patients (13.7%), distributed among 10 genotypes composed of two nonresponsive alleles, were not considered as candidates for BH_4_ loading test. Nevertheless, eight (7.8%) patients from eight genotypes of unknown responsiveness to BH_4_, composed of two nonresponsive alleles, and 13 patients (12.7%), from ten genotypes estimated not to respond to BH_4_, with just one responsive allele, can also be considered as noncandidates for BH_4_ loading test. The usual 24 h BH_4_ loading test may not be adequate for these 35 patients as they have a higher probability of being slow responders and need a longer period of time for this BH_4_ slow‐responsiveness to be observed.

## 4 DISCUSSION

A high degree of discordance (51.7%) was noticed in genotype‐phenotype correlations assessed by the arbitrary assigned values (AV) phenotype predictive system of Guldberg et al. (1998), modified by data from other researchers (Pey et al., 2003; Trunzo et al., 2016), and, for some mutations, by our own assessment. Guldberg et al. (1998) found a much lower degree of discordance (circa 20%). There could be several reasons for this finding, but it seems that the high frequency of the so‐called “moderate” mutations, p.R261Q, p.V388M, and p.I65T is at the core of this high discordance.

Taking into account the 48 patients with genotypes containing p.R261Q, p.V388M, or p.I65T mutations and whose AV sum could be calculated, a higher discordance was reached—64.6%, significantly different from the discordance found in 41 patients with other genotypes—36.6% (*z* = 2.6349, *p* = 0.0084).

Functionally hemizygous genotypes and even homoallelic genotypes containing the catalytic domain missense mutation p.R261Q, had an erratic correlation to phenotype. The seven heteroallelic genotypes containing a null mutation and p.R261Q (functionally hemizygous genotypes) had different outcomes: six classic, three moderate, and three mild PKU individuals (Table 2). Even each of the three p.R261Q homozygous patients had a different phenotype: classic, moderate, and mild. This metabolic phenotypic variability is evident in the BIOPKU database where, of the 205 homozygous patients for the p.R261Q mutation, 72 (35.1%) presented a mild PKU phenotype, while 133 (64.9%) presented a classic PKU phenotype. Kayaalp et al. (1997) attributed this phenotypic variability to a possible negative complementation of this allele in vivo, which is not apparent in in vitro studies of residual activity. However, as this variability is found in both homoallelic and functionally hemizygous genotypes, it is improbable that it is a consequence of negative allelic complementation exclusively. Danecka et al. (2015) postulated that some genotypes that involved p.R261Q (p.[R261Q]; [R261Q], p.[R261Q];[R408W], p.[R261Q];[Q355_Y356insGLQ]) had better metabolic PAH function at higher Phe concentrations. Thus, individuals with these genotypes exhibit higher Phe tolerance when their plasma Phe levels are a little above the usual therapeutic threshold. However, it is less clear how this phenomenon could at least in part explain the significant phenotypic variability found in studies like ours that classified phenotypes on the basis of pretreatment Phe levels and not Phe tolerance.

On the other hand, the genotypes containing the most frequent mutation, p.V388M—also a catalytic domain missense mutation, presented a trend toward a more severe phenotype. One p.V388M homozygous patient exhibited a classic phenotype. The nine functionally hemizygous genotypes harboring p.V388M were distributed among seven classic, five moderate, and four mild PKU patients (Table 2). This shift toward a phenotype more severe than expected by the AV system is documented in the BIOPKU database: of the 28 homozygous patients for the p.V388M mutation, 14 (50%) presented the mild and 14 (50%) the classic phenotype. We could find information in the database for seven of the nine p.V388M functionally hemizygous genotypes found in our study—three of them were reported exclusively in classic PKU patients, three in classic and mild, and only one was exclusively reported in mild phenotype patients. Other authors have also described this trend toward more severe phenotypes (Rivera et al., 2011; Santos et al., 2010). An explanation for this trend is still needed, as conflicting results regarding the variant protein V388M enzyme kinetics have been reported (Leandro, Rivera, Lechner, de Almeida, & Konecki, 2000; Staudigl et al., 2011).

Five of the six functionally hemizygous patients carrying p.I65T, a regulatory domain missense mutation, presented the classic, and just one the mild phenotype (Table 2). The distribution of functionally hemizygous individuals harboring p.I65T among different phenotype classes was already identified in the classical work of Guldberg et al. (1998). These authors speculated that a mechanism similar to the aforementioned for p.R261Q was responsible for the phenotypic diversity of functionally hemizygous genotypes containing p.I65T, but this has not been confirmed by more recent studies (Danecka et al., 2015; Staudigl et al., 2011). Moreover, it is important to note that of the 34 homozygous patients for mutation p.I65T reported in the BIOPKU database, 25 (73.5%) presented a mild phenotype but nine (26.5%) had a classic phenotype.

Sarkissian et al. (2012) analyzed the genotypes of patients that participated in the clinical trials of sapropterin, a synthetic pharmacological form of BH_4_ (6*R*‐L‐*erythro*‐5,6,7,8 tetrahydrobiopterin dihydrochloride). There are some inconsistencies between these authors’ findings and those reported at the BIOPKU database and by Zurfluh et al. (2008) based on BH_4_ loading tests (Tables 1 and 2). Sarkissian et al. (2012) reported p.V388M as an unresponsive mutation, whereas in the BIOPKU database, of the 14 homozygous patients for the p.V388M mutation tested for BH_4_ responsiveness, eight (57.14%) were responsive and two (14.29%) were slow responders. Zurfluh et al. (2008) also described it as a responsive mutation. Of the four genotypes involving p.V388M described by Sarkissian et al. (2012), three are reported at the BIOPKU database: p.[V388M];[E390G]—all 11 patients tested were BH_4_ responsive; c.[441+5G>T];p.[V388M]—one (14.3%) responder, and two slow responders (28.6%) out of seven patients tested; p.[R158Q];[V388M]—one slow responder (50%) out of two patients tested. Only the first genotype, p.[V388M];[E390G], was also reported as responsive in sapropterin clinical trials, according to Sarkissian et al. (2012). Among Ibero‐American patients tested by BH_4_ loading tests, p.V388M appeared to be at least ambiguously responsive—of the three Spanish patients homozygous for this mutation reported by Aldamiz‐Echevarria et al. (2016), one was BH_4_ responsive, and in Brazil, Nalin et al. (2011) reported one homozygous patient for p.V388M, and he was BH_4_ responsive.

Inconsistencies were also found in genotypes involving “mild” alleles. Four of the five heteroallelic functionally hemizygous genotypes involving the mutant alleles p.L48S, p.R68S, and p.L249F were observed in four classic PKU patients, and in just one patient with a moderate phenotype. Genotype‐phenotype inconsistencies have been formerly reported especially for genotypes harboring the mutation p.L48S (Danecka et al., 2015; Guldberg et al., 1998; Kayaalp et al., 1997; Pey et al., 2003). According to Danecka et al. (2015), the genotype p.[L48S];[Q355_Y356insGLQ], and other genotypes not found in our patients, present high PAH residual enzyme activity over a narrow range of Phe concentrations. Therefore, a behavior opposite to that found in p.R161Q genotypes is expected: individuals with these genotypes might exhibit lower Phe tolerance when their plasma Phe levels are above the usual therapeutic threshold.

The mutation c.1199+17G>A (rs62508613) found in four genotypes, three of them with null mutations, was clearly linked to milder phenotypes—moderate/mild PKU or even MHP. There are few reports of this mutation in the literature. Acosta, Silva, Carvalho, and Zago (2001) were the first to describe it in a moderate PKU patient with the genotype p.[R261Q];c.[1199+17G>A]. There are only six individuals reported at ClinVar (Variation ID 102555), four of them the aforementioned patients from our study. Our findings give strong support to a high residual PAH activity for these genotypes, thus conferring a mild phenotype. Nevertheless, the activity of c.1199+17G>A must be confirmed in vitro in eukaryotic cell systems.

Another factor that may be in part responsible for the high genotype‐phenotype discordance in our patients is the inclusion of the moderate phenotype category. If the BIOPKU database were used for phenotype prediction of 45 genotypes from 66 patients in our sample, the discordance would decrease to 19.7%; and if both prediction systems, the BIOPKU database and the AV system, were accepted as valid, the genotype‐phenotype discordance for 91 patients distributed among 67 genotypes would fall from 51.7% to 23.1%. The BIOPKU database uses the allelic phenotype value for predicting the metabolic phenotype of PAH variants and genotypes (Garbade et al., 2018). Nevertheless, the “predicted” phenotypes from the BIOPKU database of Table 2 were the reported phenotypes of the patients tabulated in that database. The BIOPKU database patient tabulation employs pretreatment blood Phe levels to categorize three phenotype categories—classic and mild PKU, and MHP, omitting the moderate PKU category. This simplification and the recognition of an overlapping range in blood Phe levels between mild PKU and classic PKU may have produced a reduction in genotype‐phenotype discordance when BIOPKU database phenotype tabulation data were employed.

The method used in phenotype categorization in our study, pretreatment Phe levels, deserves some concern also. We found a moderate correlation between predicted PRA and the inverse of pretreatment Phe levels (*r* = 0.456, *n* = 80). Earlier work, summarized by Enns et al. (1999), also found this correlation. But those authors themselves did not corroborate it. Rivera et al. (2000) obtained an even higher inverse correlation among early‐treated patients with PKU from Portugal (*r *= −0.773, *n* = 37). Although this literature is rather old, its focal point—the use of pretreatment Phe levels for phenotype definition, is still pertinent nowadays. Pretreatment Phe levels are still considered indispensable for phenotyping PKU as they permit classifying patients in the neonatal period (Blau, Hennermann, Langenbeck, & Lichter‐Konecki, 2011). They are extensively used for a three‐phenotype class tabulation of PKU patients in the BIOPKU database. Nevertheless, pretreatment Phe levels are especially dependent on the timing of newborn screening and on the diet the neonate was receiving at the time of blood sampling (Blau et al., 2011). Early hospital discharge after birth, and specimen collection for newborn screening <3 days of life, might have as a consequence the classification of PKU patients in milder categories. This was not an important issue in our study as the mean age at newborn screening specimen sampling for our early‐treated patients (95 out of a total of 102 patients) was 19 days.

Phe tolerance is a dependable alternative parameter for phenotyping PKU (Guldberg et al., 1998). It corresponds to the maximum amount of Phe an individual can consume and still maintain blood Phe levels within the therapeutic target range. Although Phe tolerance may be predictable already in 2‐year‐old infants, it is usually determined in children ≥5 years of age. Moreover, Phe tolerance may be difficult to determine under nonstandardized conditions, as prescribed Phe intake often is much lower than the actual Phe intake (van Wegberg et al., 2017). Another caveat for using Phe tolerance for phenotype categorization is modulation by target plasma Phe levels that can lead to some inconsistencies (Danecka et al., 2015; Guldberg et al., 1998).

*PAH* genotyping and genotype‐phenotype association studies may be employed as a screening tool for BH_4_ responsiveness, considering the high cost of the drug sapropterin—annual costs for a 29 kg patient were estimated at Can$24,000–Can$72,000 (CADTH Common Drug Reviews, 2017), especially in low‐resource settings, like Brazil (National Committee for Technology Incorporation—CONITEC, 2018) and China (Zhu et al., 2017). Additionally, the accessibility to the drug is limited due to the decision of several health technology assessment agencies not to reimburse it, including the National Committee for Technology Incorporation (CONITEC) recommendation to fund it through the Brazilian Public Health System—SUS only for women in the preconception period and during pregnancy (National Committee for Technology Incorporation—CONITEC, 2018). The importance of the complete *PAH* genotype as a selection criterion for offering BH_4_ loading test and for estimating BH_4_ responsiveness has similarly been established in high income countries (Jeannesson‐Thivisol et al., 2015; Karacic et al., 2009).

A clinically meaningful definition of BH_4_ responsiveness is widely accepted as the observation of a 20%–30% reduction in blood Phe concentration (Blau, 2008; Levy, Burton, Cederbaum, & Scriver, 2007). Several protocols have been employed to demonstrate this reduction, as there is no consensus regarding the best BH_4_ overload test (Giugliani et al., 2011). The sequential first clinical trials of sapropterin—Burton et al. (2007), Levy, Milanowski, et al. (2007) and Lee et al. (2008), defined responsiveness as a ≥30% reduction in Phe blood concentration relative to baseline values at the end of 8 days, but while the first two studies used a dose of sapropterin of 10 mg kg^−1^ day^−1^, Lee et al. (2008) evaluated daily doses of 5, 10, and 20 mg/kg to demonstrate a dose‐response relationship in the reduction in basal Phe levels. All three studies considered the same population: patients nonadherent to the recommended dietary intake of Phe, age ≥8 years old, presenting basal Phe levels ≥8 mg/dl. Trefz et al. (2009) confirmed that a dose of 20 mg kg^−1^ day^−1^ of sapropterin for 8 days was able to detect a higher response rate versus 5 mg kg^−1^ day^−1^ and 10 mg kg^−1^ day^−1^, but still maintained a ≥30% reduction in Phe blood concentration between Day 1 and Day 8 to define responsiveness.

As these multiple administrations of sapropterin and extension of the test to up to 8 days in the clinical trials are more expensive and definitely not practical for the initial screening (Blau, 2008), the BH_4_ loading test, known previously to these trials, standardized as a 24‐hr protocol after a single oral dose of 20 mg/kg of BH_4_ or sapropterin, was shown to be able to detect most responsive patients, using a cutoff of ≥30% reduction in Phe blood basal concentration (Blau & Erlandsen, 2004; Fiege & Blau, 2007). The necessity to detect a small fraction of patients, so‐called “slow responders,” which needed a repeated administration of the drug, led some PKU centers in Europe to use a 48‐hr protocol with two consecutive BH_4_ administrations of 20 mg/kg (Fiege et al., 2005; Heintz, Cotton, & Blau, 2013; Scala et al., 2015). Meanwhile, in the USA, protocols involving multiple administrations of sapropterin up until 1 month of therapy are still preferred to define BH_4_‐responsiveness (Singh & Quirk, 2011; Utz et al., 2012).

*PAH* genotyping is a useful complementary tool to BH_4_ loading test, as it can help reevaluate potential BH_4_ responsiveness misclassifications (Heintz et al., 2013; Quirk, Dobrowolski, Nelson, Coffee, & Singh, 2012). It is particularly useful in the situation of apparently responsive patients in BH_4_ loading tests that eventually do not present significant increase in baseline dietary Phe tolerance and decrease in metabolic formula needs (Quirk et al., 2012).

There are also important features to be considered from genotypes when deciding which BH_4_ loading test to be used in our particular patient population. The usual 24 or 48‐hr BH_4_ loading test may not be adequate for patients that have two nonresponsive alleles or a nonresponsive genotype as they have a higher probability of being slow responders and need a longer period for this BH_4_ slow‐responsiveness to be observed (Zhu et al., 2017). A total of 35 (34.3%) patients in this study could be included in this category.

Excluding the patients having genotypic combinations of unknown responsiveness to BH_4_, 46.2% of our patients were estimated as potential or probable BH_4_‐responders. A small‐scale study from Brazil, encompassing 18 patients, found six (33.3%) BH_4_‐responders (Nalin et al., 2011). As the prevalence of BH_4_ responsiveness has been variable in different studies (Somaraju & Merrin, 2015), we cannot make any assertion concerning the probability of the figures from our *PAH* genotyping study and Nalin et al. (2011) combined Phe and BH_4_ loading test study to be confirmed in future BH_4_ loading tests of our PKU population.

## CONFLICT OF INTEREST

Dr. Vieira Neto reports two public grants from Coordination for the Improvement of Higher Level Personnel (Capes) of the Ministry of Education, Brazil, and private grants from FBM Pharmaceutical Industry Ltd., Anápolis, Goiás, Brazil, and from Danone Ltd., São Paulo, Brazil, during the conduct of the study.
